# Comparative analysis of *Mycobacterium avium* subsp. *paratuberculosis* isolates from cattle, sheep and goats by short sequence repeat and pulsed-field gel electrophoresis typing

**DOI:** 10.1186/1471-2180-8-204

**Published:** 2008-11-25

**Authors:** Iker Sevilla, Lingling Li, Alongkorn Amonsin, Joseba M Garrido, Maria V Geijo, Vivek Kapur, Ramón A Juste

**Affiliations:** 1 Instituto Vasco de Investigación y Desarrollo Agrario (NEIKER), Berreaga, 1, 48160 Derio, Bizkaia, Spain; 2 Departments of Microbiology and Biomedical Genomics Center, University of Minnesota, MN 55108, USA

## Abstract

**Background:**

*Mycobacterium avium *subsp. *paratuberculosis *(Map) causes the chronic enteritis called paratuberculosis mainly in cattle, sheep and goats. Evidences that point out an association between Map and Crohn's Disease in humans are increasing. Strain differentiation among Map isolates has proved to be difficult and has limited the study of the molecular epidemiology of paratuberculosis. In order to asses the usefulness of the PCR based short sequence repeat (SSR) analysis of locus 1 and locus 8 in the epidemiological tracing of paratuberculosis strains we here compare for the first time the results of SSR and *Sna*BI-*Spe*I pulsed-field gel electrophoresis (PFGE) typing methods in a set of 268 Map isolates from different hosts (cattle, sheep, goats, bison, deer and wild boar).

**Results:**

A total of nineteen different multi-locus SSR (SSR1_SSR8) types were identified amongst the 268 isolates compared to the 37 multiplex profiles differentiated by the *Sna*BI-*Spe*I PFGE. SSR type 7_4 was the predominant genotype (51.2% of all isolates and 54.3% of cattle isolates), but combined with PFGE results the abundance of the most prevalent genotype (7_4&{2-1}) dropped down to 37.7%. SSR types 7_3 and 14_3 were significantly spread amongst isolates recovered from small ruminants. The comparison of SSR1_SSR8 and *Sna*BI-*Spe*I PFGE typing of these isolates has shown that both methods perform at similar discriminatory level. These were 0.691 and 0.693, respectively for SSR and PFGE as indicated Simpson's Index of Diversity, and 0.82 when calculated for combined SSR and PFGE genotypes. Overall, SSR1_SSR8 analysis seemed to detect higher levels of within-farm strain diversity and seemed to give higher year-related information. Combination of both typing methods revealed 20 multi-type farms out of the 33 bovine farms studied with more than one isolate.

**Conclusion:**

The particular SSR and PFGE typing approaches described here are in general agreement but they showed some discrepancies that might reflect differing evolutionary processes of Map strains. Both methods are able to reciprocally complement their results and neither should be replaced with the other if sufficient material and time is available. Overall, the results of our comparative analyses suggest that, based on current methodologies available, a combined approach that includes SSR and PFGE seems to provide the highest level of discrimination for Map strain typing with meaningful epidemiological information.

## Background

*Mycobacterium avium *subsp. *paratuberculosis *(Map) is the causative agent of paratuberculosis, a chronic digestive disease affecting mainly bovine, ovine, caprine and cervine livestock. Although the aetiology of Crohn's Disease has been subject of strong controversy [1,2], recent information seems to confirm an association between Map and this chronic human disease [3,4]. This underlines the increasing interest the research of Map has gained during last years due to the worldwide distribution of paratuberculosis, to the economic losses attributed to this disease [5,6], and to the presence of viable bacteria in products ready for human consumption [7-10] as a potential hazard in relationship with human inflammatory bowel disease. Successful control strategies require a good understanding of the epidemiology of a disease. Strain differentiation is a useful tool in epidemiological studies of many pathogenic bacteria. But previous investigations have revealed a relative lack of genetic diversity amongst Map isolates (reviewed in references [11,12]). Combined with the slow growth of the organism in pure culture, strain differentiation among isolates has proved to be difficult and has limited the study of the molecular epidemiology of paratuberculosis. PCR based methods can interestingly reduce the amount of bacteria and time required for Map strain typing. We here compare for the first time a set of 268 isolates from different hosts (cattle, sheep, goats, bison, deer and wild boar) that have been previously characterized for IS*1311 *PCR-restriction endonuclease analysis and *Sna*BI-*Spe*I pulsed-field gel electrophoresis (PFGE) patterns [12] with the more recently described short sequence repeat (SSR) analysis of locus 1 and locus 8 [13].

## Results and discussion

**Figure 1:**
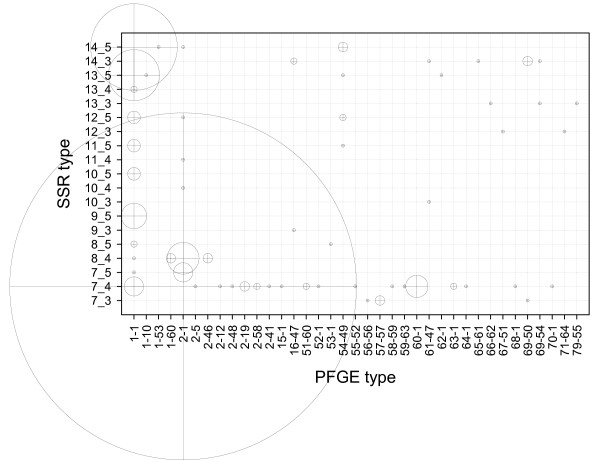
**Bubble plot showing the distribution of Map strain genotypes studied by SSR1_SSR8 and *Sna*BI-*Spe*I PFGE**. The diameter of bubbles corresponds to the number of isolates with particular SSR and PFGE types.

The results of SSR typing undertaken in the present work are summarized in Table 1. These results show that a total of nineteen different SSR1_SSR8 types were identified amongst the 268 isolates. In terms of host species distribution, there were 13 SSR types identified from cattle, 6 from sheep and 3 from goat isolates. Amongst isolates recovered from Spain, SSR type 7_4 accounted for the 54.3% of cattle isolates, while types 7_3 and 14_3 accounted for the 29% of sheep isolates each. Interestingly, amongst isolates recovered from goats, approximately the same proportion (43%) of isolates was typed as either cattle type 7_4 or sheep type 14_3. The remaining 14.3% of goat isolates were also sheep type strains and were identified as 9_3 type in SSR. Both deer and wild boar isolates belonged to the widest distributed type 7_4, in contrast they were {68-1} and {2-1} profiles in PFGE, respectively. Genetic homogeneity of Map isolates has been previously pointed out by other researchers using different typing methods [14-17]. Similarly and in agreement with our results, SSR method has demonstrated predominant type 7_4 to account for more than half the strains analyzed in previous studies [18,19]. The combination of SSR and PFGE types found in the present work made the prevalence of the most abundant genotype (7_4&{2-1}) drop down to 37.7%. None of the remaining combined types showed prevalences over 10%, except the combined type 14_5&{1-1}. The latter corresponds to the type assigned to MAP K10 strain and it was found in 10.07% of isolates under study. The amount of isolates showing particular genotypes in both techniques is graphically represented in Figure 1.

**Table 1:** SSR1_SSR8 classification of *Map *strains.

**Country**	**Region Code**	**SSR1_SSR8**	**Host sp**	**no. of isolates (%)**	**no. of farms (%)**	**IS*1311 *type**
Spain	BC, As, CL, Cat, Can, An, Ga, Ar, Ma, Na, CM	7_4	Cattle	126 (54.31)	80 (61.07)	C type
	BC, Ar, Na	7_5	Cattle	7 (3.02)	2 (1.53)	C type
	BC, Ex, CL, Can, Ga	8_4	Cattle	17 (7.33)	14 (10.69)	C type
	BC, Ar	8_5	Cattle	3 (1.29)	3 (2.29)	C type
	BC, Na	9_5	Cattle	8 (3.45)	3 (2.29)	C type
	BC	10_4	Cattle	1 (0.43)	1 (0.76)	C type
	BC, Na	10_5	Cattle	4 (1.72)	3 (2.29)	C type
	BC	11_4	Cattle	1 (0.39)	1 (0.76)	C type
	BC	11_5	Cattle	5 (2.16)	5 (3.82)	C type
	BC, Can, Cat	12_5	Cattle	7 (3.02)	7 (5.34)	C type
	BC	13_4	Cattle	2 (0.86)	2 (1.53)	C type
	As, BC, Na, Ar	13_5	Cattle	19 (8.19)	13 (9.92)	C type
	BC, Na, Ar	14_5	Cattle	32 (13.79)	22 (16.79)	C type
	BC, Ar, Na	7_3	Sheep	5 (29.41)	4 (40.0)	S type
	Ar	7_4	Sheep	1 (5.88)	1 (10.0)	C type
	BC	10_3	Sheep	1 (5.88)	1 (10.0)	S type
	BC	12_3	Sheep	2 (11.76)	2 (20.0)	S type
	BC	13_3	Sheep	3 (17.65)	3 (30.0)	S type
	BC, Na	14_3	Sheep	5 (29.41)	4 (40.0)	S type
	BC, An	7_4	Goat	3 (42.86)	2 (40.0)	C type
	CL	9_3	Goat	1 (14.29)	1 (20.0)	S type
	IB	14_3	Goat	3 (42.86)	3 (60.0)	S type
	CM	7_4	Deer	1 (100.0)	1 (100.0)	C type
	CM	7_4	Wild Boar	1 (100.0)	1 (100.0)	C type

India	Mathura	7_4	Sheep	2 (100.0)	1 (100.0)	B type
	Farah	7_4	Goat	5 (100.0)	1 (100.0)	B type

USA	Montana	7_4	Bison	3 (100.0)	1 (100.0)	B type

SSR types by region and host species. Name of SSR type: number of repeats in locus 1 (G residue) _ number of repeats in locus 8 (GGT residue). IS*1311 *PCR-REA classification of isolates is also included. Percentages in brackets are calculated according to the total number of isolates in each host species. Regions mentioned in the study are indicated as follows: An = Andalucia; Ar = Aragón; As = Asturias; BC = Basque Country; Can = Cantabria; Cat = Cataluña; CL = Castilla y León; CM = Castilla-La Mancha; Ex = Extremadura; Ga = Galicia; IB = Balearic Islands; Ma = Madrid; Na = Navarra.

Cluster analysis with both PFGE and SSR based typing methods (not shown) confirmed that Map isolates are genetically divided into the cattle type and sheep type main groups, much as has been found in other works [20-25]. The agreement between this classification and the IS*1311 *groups *C *(including the less common *B *strains in this group) and *S *confirms the utility of IS*1311 *PCR-REA as a rapid and reliable method for preliminary typing of Map isolates, as indicated by results of previous works [12,26].

While the overall discriminatory power of both methods as calculated by Simpson's index of diversity (1-D) was almost the same (0.693 for PFGE and 0.691 for SSR), comparative analysis revealed that the most abundant PFGE {1-1}, {2-1} and {54-49} profiles (30%, 48% and 2.7% of all isolates, respectively) were subdivided into 11, 7 and 4 different types, respectively. Similarly, isolates representing the most abundant SSR type 7_4 (51% of all isolates) could be subdivided into 19 different PFGE profiles as shown in Table 2. As was to be expected, the overall 1-D value raised up to 0.82 if calculated considering the abundances of combined SSR and PFGE types (i.e. 7_4&{51–60}, 7_4&{2-1} ...). Other promising methods as the mycobacterial interspersed repetitive units/variable number tandem repeats (MIRU/VNTR) seem to have a high discriminatory power as well [27,28]. Amongst isolates recovered from sheep, there was a higher discrimination with PFGE (1-D = 0.865) than with SSR (1-D = 0.775), but such a difference was not noticed in the other host species (see Table 3).

**Table 2:** Reciprocal complementation between SSR1_SSR8 and *Sna*BI-*Spe*I PFGE.

**PFGE type**	**subdivided by**	**SSR types**
{1-1}	11	7_4, 7_5, 8_4, 8_5, 9_5, 10_5, 11_5, 12_5, 13_4, 13_5, 14_5
{2-1}	7	7_4, 7_5, 8_4, 10_4, 11_4, 12_5, 14_5
{16–47}	2	9_3, 14_3
{61-47}	2	10_3, 14_3
{54–49}	4	11_5, 12_5, 13_5, 14_5
{69-50}	2	7_3, 14_3
{69-54}	2	13_3, 14_3
		
**SSR type**	**subdivided by**	**PFGE types**

7_3	3	{56-56}, {57-57}, {69-50}
7_4	19	{1-1}, {2-1}, {2–41}, {15-1}, {52-1}, {60-1}, {2–5}, {2–12}, {2–48}, {2–19}, {2–58}, {51–60}, {55-52}, {58–59}, {59–63}, {63-1}, {64-1}, {68-1}, {70-1}
7_5	2	{1-1}, {2-1}
8_4	4	{1-1}, {1–60}, {2-1}, {2-–46}
8_5	2	{1-1}, {53-1}
11_5	2	{1-1}, {54-49}
12_3	2	{67-51}, {71-64}
12_5	3	{1-1}, {54-49}, {2-1}
13_3	3	{66-62}, {79-55}, {69-54}
13_5	4	{1-1}, {1–10}, {54–49}, {62-1}
14_3	5	{16–47}, {61-47}, {65-61}, {69-50}, {69-54}
14_5	4	{1-1}, {2-1}, {1–53}, {54-49}

Number of SSR1_SSR8 types identified within each *Sna*BI-*Spe*I PFGE multiplex profile and vice versa, showing how each technique complements each other in subdividing the most prevalent types.

**Table 3:** Genetic diversity and discriminatory power of SSR1_SSR8 and *Sna*BI-*Spe*I PFGE.

	**PFGE**	**SSR**
	
**number of different types**	37	19
	**1-D value**	
	
**cattle**	0.621	0.669
**sheep**	0.865	0.775
**goat**	0.666	0.612
**Discriminatory power**	0.693	0.691
**combined**	0.817	

Estimation of the genetic diversity of isolates from Spanish cattle, sheep and goats, and the discriminatory power of both methods calculated as Simpson's Index of Diversity (1-D). Discriminatory power of the combined SSR&PFGE method was calculated taking into account all combinations found amongst Map isolates studied.

The polyclonal infection of one Holstein bull with three different strains earlier demonstrated by PFGE [12] was partially confirmed by SSR1_SSR8 typing. The isolate typed as {1-1} with PFGE was of 13_5 type by SSR, but the remaining two isolates classified as PFGE profiles {2-1} and {59-63} shared the same SSR type 7_4. None of the fingerprinting methods compared here detected any other polyclonal infections in the other three animals with more than one culture available included in the study. In general terms, SSR1_SSR8 analysis seemed to detect higher levels of within-herd strain variability than *Sna*BI-*Spe*I PFGE. With 33 bovine farms giving more than one isolate, SSR method used detected 17 farms with multi-type isolates, one farm with isolates belonging to five different types, two farms yielded isolates of four different types, another one gave isolates of 3 types, and finally 13 of these herds had isolates of two distinct SSR types (Table 4). On the other hand, PFGE typing detected up to 14 farms with multi-type isolates, three bovine herds with three different profiles and 11 herds carrying strains of two different profiles. Combination of both typing methods revealed 20 multi-type cattle farms. More than one isolate was recovered from four sheep flocks. In this case, a slightly higher level of intra-herd variability was detected by PFGE compared to SSR. Three flocks with 3 different strains were found by PFGE while SSR analysis identified two flocks with 3 different types and one flock with two.

**Table 4:** Multi-type herds and sheep flocks detected by SSR1_SSR8 and *Sna*BI-*Spe*I PFGE.

								**multi-type farm?**	
								
**Farm**	**Region**	**sp**	**Breed**	**SSR1_8 type**	**no. of isolates**	***Sna*BI-*Spe*I PFGE type**	**no. of isolates**	**SSR**	**PFGE**
**BI5**	BC	Bov	Holstein	8_4,9_5	1,2	{1-1}	3	yes	no
**BI6**	BC	Bov	Holstein	7_4,9_5,10_5,14_5	1,3,2,1	{1-1},{2-1}	6,1	yes	yes
**SS5**	BC	Bov	Holstein	8_5,11_5,13_5,14_5	1,1,3,1	{1-1},{54-49}	5,1	yes	yes
**SS15**	BC	Bov	Holstein	11_5,14_5	1,1	{1-1}	2	yes	no
**SS20**	BC	Bov	Holstein	7_4	3	{2-1},{2–19}	2,1	no	yes
**SS23**	BC	Bov	Holstein	7_4,14_5	10,1	{2-1},{54-49}	10,1	yes	yes
**SS27**	BC	Bov	Holstein	7_4,13_5	3,2	{1-1},{2-1},{59-63}	2,2,1	yes	yes
**SS28**	BC	Bov	Holstein	7_4,12_5	1,1	{2–19},{54-49}	1,1	yes	yes
**SS38**	BC	Bov	Holstein	13_5,14_5	1,1	{1-1}	2	yes	no
**SS43**	BC	Bov	Pyrenean	12_5,14_5	1,1	{1-1}	2	yes	no
**SS45**	BC	Bov	Limousin	7_4,14_5	1,1	{2-1},{54-49}	1,1	yes	yes
**SS52**	BC	Bov	Holstein	7_4,13_5,14_5	1,1,1	{1-1},{2-1}	2,1	yes	yes
**S6**	Can	Bov	Holstein	7_4,8_4	1,1	{2-1},{2–46}	1,1	yes	yes
**BU3**	CL	Bov	Holstein	7_4,8_4	2,2	{2-1}	4	yes	no
**VA1**	CL	Bov	Holstein	7_4	5	{2-1},{2–41}	4,1	no	yes
**NA1**	Na	Bov	Holstein	9_5,10_5	3,1	{1-1}	4	yes	no
**NA2**	Na	Bov	Holstein	13_5,14_5	4,9	{1-1},{1–53},{62-1}	11,1,1	yes	yes
**O4**	As	Bov	Holstein	7_4	3	{1-1},{2-1}	2,1	no	yes
**SA2**	CL	Bov	Bullfight	7_4,8_4	1,1	{2-1},{2–46}	1,1	yes	yes
**Z2**	Ar	Bov	Holstein	7_4,7_5,8_5,13_5,14_5	1,6,1,1,3	{1-1},{2-1},{53-1}	5,6,1	yes	yes
**SS53**	BC	Ov	Latxa	7_3,10_3,14_3	1,1,1	{61-47},{69-50},{69-54}	1,1,1	yes	yes
**SS54**	BC	Ov	Latxa	12_3,13_3,14_3	1,1,1	{67-51},{79-55},{69-50}	1,1,1	yes	yes
**SS55**	BC	Ov	Latxa	12_3,14_3	1,2	{61-47},{69-50},{71-64}	1,1,1	yes	yes

Farms with more than one isolate giving at least two different strain types in SSR and/or PFGE typing. SSR detected 17 multi-type bovine herds and 3 multi-type sheep flocks. In the other hand, PFGE identified 14 and 3 multi-type farms, respectively. Farm names indicate the name of the province (within a region) they belong to: BI = Bizkaia (BC); SS = Gipuzkoa (BC); S = Cantabria (Can); BU = Burgos (CL); VA = Valladolid (CL); NA = Navarra (Na); O = Asturias (As); SA = Salamanca (CL); Z = Zaragoza (Ar). Other abbreviations: As = Asturias; BC = Basque Country; Can = Cantabria; CL = Castilla y León; Na = Navarra; Bov = bovine; Ov = ovine.

A previous work suggested an apparent relation between particular G residue repeat alleles and host species in SSR analysis [29]. In the present study the previously described alleles 7G to 14Gs have been found, but 8Gs, 9Gs and 11Gs are almost restricted to isolates obtained from cattle. Interestingly, there seems to be a strong link between GGT residue alleles and host species. Thus, allele 3GGTs has been detected in all sheep (except one) and in 67% of goats while all cows analyzed were infected with strains showing 4 or 5GGTs repeats. Possession of 3GGT allele resembles possession of a cytosine at base pair position 223 that can be found in all copies of the IS*1311 *gene of typical sheep (*S*) type strains of Map [30].

The study of SSR1_SSR8/*Sna*BI-*Spe*I PFGE combined profiles from herds giving more than one isolate according to the date of cultures demonstrated the reliability and usefulness of these techniques for epidemiological tracing of paratuberculosis cases. Eleven Holstein farms giving at least two isolates from different years were identified. The SSR method appeared to give slightly higher year-related information. As shown in Table 5, strain type changed along the years during the follow-up period in farms SS5, SS27, SS28, SS38 and SS52 (the meaning of letters used to name farms under study is given in Table 5). On the contrary, farms BI2, BI9, HU1, LE1 and NA2 maintained the same strain types year after year. Herd SS23 showed an intermediate situation since it yielded two types in the first year, but maintained one of them afterwards. Further conclusions cannot be suggested due to a lack of information. A strain variation percentage was calculated for each of these farms dividing the number of different strain types minus one by the total number of isolates recovered minus one. Thus a 100% strain variation was observed for the first 3 farms while the last four showed no variation (Table 5). SS5, SS27, NA2 and SS23 showed intermediate strain variations of 60, 50, 25 and 10%, respectively. Collectively, our results indicate that SSR1_SSR8 analysis, helped by *Sna*BI-*Spe*I PFGE where possible, can offer very valuable epidemiologic information and indicate the existence of three models of strain type change in infected populations: stable, variable and intermediate. No obvious difference in the incorporation of new animals to the herd was observed between the different types of farms but the information on other management factors was very scarce. However, for the first time it has been shown that epidemiological patterns can vary according to cattle population and time. This observation requires further research by broadening the number of farms and extending the period of observation, as well as recording factors that might influence the strain shifting and determine its consequences in terms of severity of the disease, control measures effects and bacteria sources and reservoirs.

**Table 5:** Circulation of Map strains in some bovine herds along time.

	**SSR1_SSR8&*Sna*BI-*Spe*I PFGE profiles identified in different years (number of isolates in brackets)**					
	
**Farm**	**2000**	**2001**	**2002**	**2003**	**2004**	**2005**
**SS28**	12_5/{54-49} (1)	-	-	7_4/{2–19} (1)	-	-
**SS38**	-	-	13_5/{1-1} (1)	14_5/{1-1} (1)	-	-
**SS52**	-	-	7_4/{2-1} (1)	13_5/{1-1} (1)	14_5/{1-1} (1)	-

**SS5**	-	8_5/{1-1} (1)	11_5/{54-49} (1) 13_5/{1-1} (3)	14_5/{1-1} (1)	-	-
**SS27**	-	-	13_5/{1-1} (2)	7_4/{2-1} (2) 7_4/{59-63} (1)	-	-
**NA2**	-	-	-	-	13_5/{1-1} (1) 14_5/{1-1} (1)	13_5/{1-1} (2) 13_5/{62-1} (1) 14_5/{1-1} (7) 14_5/{1-53} (1)
**SS23**	-	7_4/{2-1} (1) 14_5/{54-49} (1)	7_4/{2-1} (8)	-	7_4/{2-1} (1)	-

**BI2**	-	-	7_4/{2-1} (1)	7_4/{2-1} (2)	-	-
**BI9**	-	-	7_4/{2-1} (1)	-	7_4/{2-1} (2)	7_4/{2-1} (2)
**HU1**	-	-	7_4/{2-1} (1)	-	7_4/{2-1} (1)	-
**LE1**	-	7_4/{2-1} (3)	7_4/{2-1} (4)	-	-	-

New strains turning up and within-herd spread of specific strains during time in some bovine Holstein farms as assessed by SSR/PFGE analysis combination. Strain variation along time seems to indicate three different epidemiologic situations: Farms with 100% strain variation (variable), farms with intermediate strain variation (intermediate) and farms carrying always the same types (stable).

## Conclusion

These independent typing methods are in general agreement. However, they showed significant discrepancies indicating that each one might reflect differing evolutionary processes of Map strains. Since both SSR1_SSR8 and *Sna*BI-*Spe*I PFGE methods have the ability to reciprocally complement their results by subdividing the different genotypes identified in the other method, none of them should be used as a substitute for the other one if sufficient bacterial growth is available. Taken together, the results of our studies confirm the utility of the SSR approach as an easy and rapid method based on PCR and sequence analysis that requires only small amounts of sample to perform, compared to the big amount and good quality of DNA required for PFGE typing. The results also suggest that the addition of a third locus to SSR1_SSR8 typing may help in increasing the discriminatory power of this method. Overall, the results of our comparative analyses suggest that, based on current methodologies available, a combined approach that includes IS*1311 *PCR-REA, SSR and PFGE provides the highest level of discrimination for Map strain characterization. However, in practical terms, the use of IS*1311 *PCR-REA is not equivalent to the other two since it only provides broad group classification. The choice between PFGE and SSR, however, will be defined for the technical simplicity, lower DNA quality and quantity requirements and robustness for obtaining reliable epidemiologic information of SSR.

## Methods

DNA from 232 isolates from cattle (Spain), 19 from sheep (17 from Spain and two from India), 12 from goats (seven from Spain and five from India), one from deer (Spain), one from wild boar (Spain) and three from bison (USA) grown on Herrold's egg yolk, Lowenstein-Jensen (Biomedics, Madrid, Spain) and/or Middlebrook media (Becton, Dickinson and Company, MD, USA) with or without supplements (mycobactin J and/or OADC enrichment) used in a previous work [12] was analyzed. In the previous PFGE study mentioned above isolates were classified as cattle (*C*), sheep (*S*) or bison (*B*) strains by IS*1311 *PCR-REA and subdivided into 37 different multiplex *Sna*BI-*Spe*I PFGE profiles (the PFGE nomenclature used earlier has been changed according to the instructions of the standardized database at . In the present paper square brackets have been replaced with curly brackets to distinguish between PFGE nomenclature and literature references, except in Figure 1). DNA was purified from proteinase K pre-treated agarose plugs previously prepared for PFGE. A piece of plug was cut and introduced into a 1.5 ml tube. QIAquick PCR purification kit (Qiagen, GmbH, Germany) was used according to the instructions of the manufacturer to remove the agarose and cell debris. One μl of purified DNA was used for PCR amplification of the most discriminatory SSR loci 1 (G residue) and 8 (GGT residue) as described earlier [13]. Afterwards, PCR products were sequenced by using standard dye terminator chemistry, and the sequences analyzed on a 3700 DNA Analyzer (Applied Biosystems, Foster City, CA, USA). All chromatograms were visually inspected, and sequences edited with the EditSeq program (DNASTAR, Madison, WI, USA) and then aligned by the use of the MegAlign program (DNASTAR). The number of G repeats in locus 1 and the number of GGT repeats in locus 8 separated by one underscore was used to designate different SSR genotypes (SSR1_SSR8). A bubble type plot was generated with SigmaPlot for Windows v10 software (Systat Software, Inc., San Jose, CA, USA) in order to show the number of isolates in a SSR type versus PFGE type matrix. Simpson's Index of Diversity (1-D) was calculated as follows in order to compare the genetic diversity of isolates between host species and to asses the discriminatory power of the typing methods used:

Simpson's index of diversity = 1-D = 1-[Σ(no. of isolates with a particular genotype/total no. of isolates)^2^]

## Authors' contributions

IS and LL carried out the laboratory work, compiled and analysed information and data, and drafted the manuscript. AA, JMG and MVG helped to draft the manuscript. VK and RAJ conceived of the study, and helped to draft the manuscript. All authors read and approved the final manuscript.
